# A Comprehensive Evaluation of Graph Kernels for Unattributed Graphs

**DOI:** 10.3390/e20120984

**Published:** 2018-12-18

**Authors:** Yi Zhang, Lulu Wang, Liandong Wang

**Affiliations:** 1 State Key Laboratory of Complex Electromagnetic Environment Effects on Electronics and Information System, Luoyang 471003, China; 2 National Innovation Institute of Defense Technology, Academy of Military Science, Beijing 100071, China

**Keywords:** graph kernel, unattributed graph, time complexity, classification accuracy, graph dataset

## Abstract

Graph kernels are of vital importance in the field of graph comparison and classification. However, how to compare and evaluate graph kernels and how to choose an optimal kernel for a practical classification problem remain open problems. In this paper, a comprehensive evaluation framework of graph kernels is proposed for unattributed graph classification. According to the kernel design methods, the whole graph kernel family can be categorized in five different dimensions, and then several representative graph kernels are chosen from these categories to perform the evaluation. With plenty of real-world and synthetic datasets, kernels are compared by many criteria such as classification accuracy, F1 score, runtime cost, scalability and applicability. Finally, quantitative conclusions are discussed based on the analyses of the extensive experimental results. The main contribution of this paper is that a comprehensive evaluation framework of graph kernels is proposed, which is significant for graph-classification applications and the future kernel research.

## 1. Introduction

Graphs are important structures for information representation, in which nodes and edges respectively represent the entities and the relationships in the real world. Graph processing has been widely used in many scientific fields such as image processing [1], biochemical research [2], social network [3] and natural language processing [4]. Meanwhile, graph comparison plays a core role in data mining and target recognition in these fields. For instance, two molecules with the same chemical properties usually have similar topological structures [5]. Thus people can successfully perform a prediction for an unknown molecule via topology comparison with known ones.

It has been reported that exact graph comparison is equivalent to sub-graph isomorphism detection, which is a well-known NP-hard problem [6]. Inexact substitutions have to be explored, such as approximate graph edit distance [7], topological descriptors [8] and graph kernels.

The construction of graph kernels has been extensively investigated in the past decades. Through a well-defined kernel function, two samples in the graph space could be mapped into a real number, which represents the quantitative similarity between two graphs. Moreover, extracting the graph features explicitly is unnecessary in this procedure.

However, as stated in [9], an exact graph kernel can locate the dissimilarity between two arbitrary and non-isomorphic graphs. Thus it has the same time complexity with graph isomorphism detection, which is NP-hard but has been neither proven as NP-complete nor found to be solved by any polynomial-time algorithm. By now, all the existing graph kernels are actually inexact, paying the distinct tradeoffs among classification accuracy, applicability and runtime performance.

Unfortunately, the problem of how to make a qualitative comparison of graph kernels has not been solved in the current literature. According to [10], “There is no theoretical justification on why certain types of kernels are better than the others”. Therefore, almost all existing approaches only utilize a few real-world datasets to test the classification performance of the proposed kernels. However, this kind of weak evaluation cannot give an effective guidance when we need an optimal method for a practical graph-classification application.

In order to alleviate this problem, a comprehensive evaluation framework of graph kernels is discussed in this paper. According to the design details of the kernel method, the whole graph kernel family can be grouped in five different dimensions. We choose several popular graph kernels to perform a comprehensive evaluation. Through classification tests on plenty of real-world and synthetic datasets, these graph kernels are compared by many criteria such as classification accuracy, F1 score, runtime cost, scalability and applicability. Finally, quantitative conclusions on the graph kernel groups are discussed based on the analyses of the experimental results.

In this paper, we mainly focus on the classification evaluation for unattributed graphs, all the nodes and vertices of which have no attributes [11,12]. The reasons are twofold. Firstly, unattributed graph is more common in the real world because the attributes of entities and relationships are usually difficult and expensive to capture. Secondly, the design of kernel methods for unattributed graphs is more challenging owing to the lack of information. Some researches based on graphs with continuous or high-dimension attributes [13,14] are not considered in our evaluation.

## 2. Graph Kernel

### 2.1. Graph Definition

An unattributed graph G=(V,E) is a set of unlabeled vertices and links. $v_{1},v_{2}\ldots v_{n}\in V$ is the set of vertices. E is the adjacency matrix. If there is a link between $v_{i}$ and $v_{j}$, then $E\left(v_{i},v_{j}\right)=1$. Otherwise, $E\left(v_{i},v_{j}\right)=0$.

A graph $\hat{G}=\left(\hat{V},\hat{E}\right)$ is an (induced) subgraph of graph G=(V,E), if and only if $\hat{V}\subseteq V$ and $\forall v_{1},v_{2}\in \hat{V},\hat{E}\left(v_{1},v_{2}\right)=E\left(v_{1},v_{2}\right)$. For an unattributed graph, a subgraph is also called as a pattern of a graph.

A (sub)graph G=(V,E) is isomorphic to $H=\left(V^{\prime },E^{\prime }\right)$, if there exists as least one bijective function $f:V\to V^{\prime }$ so that $\forall v_{1},v_{2}\in E\Leftrightarrow \left(f_{v_{1}},f_{v_{2}}\right)\in E^{\prime }$. We denote it as G≅H.

A graph alignment $\Phi^{G}=\left(G^{\alpha },\vartheta \right)$ is defined by a set of several subgraphs $G^{\alpha }\left(\alpha =1,\ldots ,p\right)$ and a special order of the nodes $\left\{v_{1}^{\alpha },\ldots ,v_{k}^{\alpha }\right\}$ in each subgraph [15]; this joint order is denoted by ϑ. Therefore $\Phi^{G}=\Phi^{H}$ means that the two graphs G and H are aligned.

### 2.2. Kernel Method

**Figure 1:**
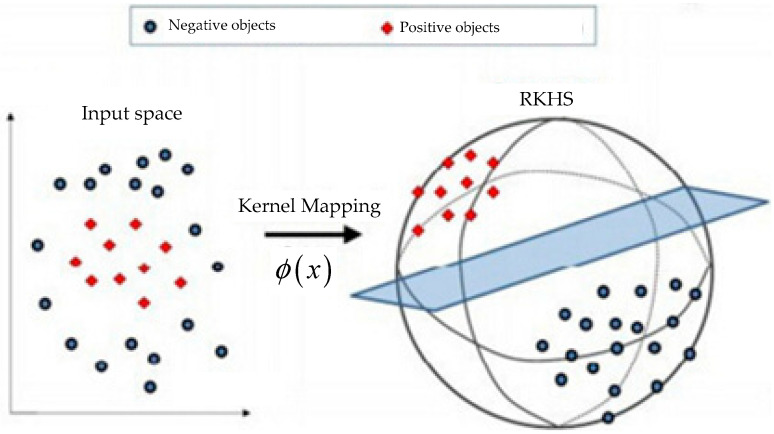
Example of kernel method.

Kernel method is an important concept in machine learning. Through a mapping ϕ from the input space S into the reproducing kernel Hilbert space H (RKHS), the kernel function is defined as:

(1) $$k\left(x,x^{\prime }\right)={\langle \phi \left(x\right),\phi \left(x^{\prime }\right)\rangle }_{H}$$

where $x,x^{\prime }\in S$ and ${\langle \cdot ,\cdot \rangle }_{H}$ is the inner product operation in RKHS. An illustration of kernel method is shown in Figure 1. The most obvious advantage of graph kernel is that a problem which cannot be linearly separated is changed into a linear separable problem through a kernel mapping.

Specifically, we call the method as graph kernel if the input space is the graph space. From Equation (1), it is clear that via a feature extraction method ϕ, graphs can be mapped into vectors in a RKHS, and the inner product of two vectors can represent the graph similarity.

Usually, the graph similarity can be computed directly, without the explicitly definition of the feature extraction method ϕ. In the real world, it is quite difficult to compute an explicit embedding representation of structured data. Therefore, compared to topology descriptors and other graph comparison methods, graph kernel could achieve a smart and accurate similarity measurement because of the implicit feature extraction. In the field of machine learning, it has been witnessed that graph kernels can bridge the gap between graph-based data and a large group of kernel-based machine learning algorithms including support vector machines (SVM), kernel regression, and kernel principle component analysis (kPCA) [9].

According to the Mercer’s theorem [16], a valid graph kernel must be symmetric and positive semi-definite (p.s.d.):

1. Symmetric. Obviously, for two graphs $G_{A}$ and $G_{B}$, $k\left(G_{A},G_{B}\right)=k\left(G_{B},G_{A}\right)$.
2. p.s.d. For a dataset with n graphs, any finite sequences of graphs $g_{1},g_{2},\cdots g_{n}$ and any choices of arbitrary real numbers $c_{1},c_{2},\cdots c_{n}$, we have $\sum_{i=1}^{n}\sum_{j=1}^{n}k\left(g_{i},g_{j}\right)c_{i}c_{j}\geq 0$, i.e., all the eigenvalues of the kernel matrix $K_{n\times n}=\left[k\left(g_{i},g_{j}\right)\right]$ for the dataset are nonnegative.

Note that although the learning ability of valid graph kernels has been proven, there is no theoretical research which demonstrates that invalid graph kernel could not support the learning algorithms. Actually, there exist discussions of how similarities (i.e., non-p.s.d. kernels) can support learning [17,18]. Therefore, some graph kernels are not proved to be p.s.d. in the literatures.

### 2.3. Kernel Groups

In the literature, the existing graph kernels are usually designed based on the distinct topological analysis of graph structure. According to five different dimensions of kernel design details, the whole family of graph kernels can be categorized as follows:

1. Framework: R-convolution kernels vs. Information theoretic kernels

In 1999, Haussler proposed R-convolution kernels [19], which discover the relationship between graph similarity and the appearances of same or similar substructures of two graphs. As the formal definition in [20], for two graphs $G_{1}$ and $G_{2}$, $\left\{S_{1;1},\cdots ,S_{1;n_{1}},\cdots ,S_{1;N_{1}}\right\}$ and $\left\{S_{2;1},\cdots ,S_{2;n_{2}},\cdots ,S_{2;N_{2}}\right\}$ are sub-graph sets of $G_{1}$ and $G_{2}$ respectively. A standard R-convolution kernel K for these two graphs is defined as:

(2) $$K\left(G_{1},G_{2}\right)=\sum_{n_{1}=1}^{N_{1}}\sum_{n_{2}=1}^{N_{2}}\delta \left(S_{1;n_{1}},S_{2;n_{2}}\right)$$

where δ denotes a Dirac kernel shown as:

(3) $$\delta \left(S_{1;n_{1}},S_{2;n_{2}}\right)=\{\begin{array}{l} 1,\;\mathrm{if}\;S_{1;n_{1}}≅S_{2;n_{2}}\\ 0,\;\mathrm{otherwise}\; \end{array}$$

where $S_{1;n_{1}}≅S_{2;n_{2}}$ indicates the substructure $S_{1;n_{1}}$ is isomorphic (or approximately isomorphic) to $S_{2;n_{2}}$.

So far, most existing kernels [9,10,21,22,23] belong to this group. Intuitively, two similar graphs should have many common substructures. However, the main drawback of the R-convolution kernels is that they neglect the relative locations of substructures. This is because R-convolution kernels cannot establish reliable structural correspondences among substructures [21]. Meanwhile, the teeter-totter problem and the complexity issue of the graph decomposition challenge the development of R-convolution kernels [6].

Recently, Bai et al. utilized information theory methods to compute the probability distribution diffusion of two graphs as the similarity measurement [20,24,25,26,27,28]. By mapping all the data points of the input space into a fitted distribution in a parametric family S, a kernel for the distributions can be defined. This group of kernels for the data points in terms of distributions can be automatically induced in the original input space. Therefore, this framework provides us an alternative way of defining kernels that maps graphs into a statistical manifold. In real-world applications, these kernels outperform the linear kernels using SVM classifiers. Some of them create a bridge between kernel methods and information theory, and thus have an information theoretic interpretation. These methods are called information theoretic kernels. However, its high computational complexity of the information entropy is the bottleneck of this group.

2. Graph Pre-processing: Aligned Kernels vs. Unaligned Kernels

Graph alignment can locate the mapping relationship between the node sequences of two graphs. It is a pre-processing procedure for the original graphs. Common substructures will be pinned in the same position in these two graphs after the alignment. Through assigning parts of one object to parts of the other, the most similar parts of the two objects can be found out. Finding such a bijection is known as the assignment problem and well-studied in combinatorial optimization. This approach has been successfully applied to graph comparison, e.g., in general graph matching as well as kernel-based classification. In contrast to convolution kernels, assignments establish structural correspondences, thereby alleviating the problem of diagonal dominance at the same time. And then it can achieve an accurate similarity computation without false positive. The research on optimal assignment kernels was reported in [29], in which each pair of structures is aligned before comparison. However, the similarities derived in this way are not necessarily positive semi-definite (p.s.d.) and thus do not give rise to valid kernels, severely limiting their use in kernel methods [30]. Kriege et al. discussed the condition for guaranteeing an optimal assignment kernel to be positive semi-definite [31].

The performance of the aligned kernel depends on the alignment accuracy. During graph alignment, every node/subgraph in a graph could only map to one node/subgraph in another graph, which will lead to information loss. Therefore, unaligned kernels are more common in the literature.

3. Matching Pattern: Global kernels vs. Local kernels

A novel choice of matching pattern is usually the core of designing a new graph kernel. The similarity computation of a kernel mainly depends on the exploration of the matching patterns.

Most existing graph kernels belong to the group of local kernels, which focus on local patterns of data structure, such as walks [22], paths [23], sub-trees [21] and limit-sized sub-graphs [10]. For example, the random walk kernel will embed graph into a feature space, in which the feature vector consists of walk numbers with different lengths.

A few studies changed to explore the global characteristics of graphs such as the Lovász number [32] and the whole probability distribution [11]. The kernels based on these methods are called global kernels. In this kernel group, all the members directly obtain the similarity among graphs based on the whole structure information. Therefore, the graph decomposition is not necessary.

In the most recent research, some hybrid patterns were utilized to design a kernel [33]. Because graph matching is a special case of sub-graph matching, the kernels based on graph matching are allocated in the group of local pattern in this paper.

4. Computing Models: Quantum kernels vs. Classical kernels

Quantum computation differs from its classical counterparts. It can store, process and transport information using the peculiar properties of quantum mechanics such as the superposition and the entangled state. These properties result in an exponential increase of the dimensionality of the state-space, which is the basis of the quantum speedup.

As the quantum counterpart of random walk, quantum walk [34] becomes a novel computing model to analyze graph structures. Because of the amazing properties of quantum parallel and quantum interference, the quantum amplitude of quantum walks on a graph could represent more complex structural information. For kernels based on the discrete-time edge-based quantum walk (DTQW) [12,35] or the concrete-time node-based quantum walk (CTQW) [11,21], all of them are called quantum kernels, and the others are called classical kernels. In fact, quantum walk is the only method used to design the quantum kernels in literature.

Here we use DTQW as an instance. The discrete-time quantum walk is the quantum counterpart of the classical random walk. We denote the state space of DTQW as E, which is the edge set of a graph. And a general state of the quantum walk is:

(4) $$|\varphi \rangle =\sum_{(u,v)\in E}\alpha_{uv}|uv\rangle$$

where the quantum amplitudes $\alpha_{uv}$ are complex. Using the Grover diffusion matrix, the entries of the transition matrix U is shown as follows:

(5) $$U_{(u,v),\left(w,x\right)}=\{\begin{array}{cc} \frac{2}{d_{x}}-\delta_{ux}, & v=w\\ 0, & otherwise \end{array}$$

where $U_{(u,v),\left(w,x\right)}$ shows the quantum amplitude for the transition |uv〉→|wx〉, $d_{x}$ means the vertex degree and $\delta_{ux}$ is the Kronecker delta, i.e., $\delta_{ux}=1$ if u=x, otherwise $\delta_{ux}=0$.

Different from random walk where the probability propagates, what propagates during quantum walk is the quantum amplitude. Therefore, the quantum interference will happen between two crossing walks. In this paper, we only consider quantum computing as a novel computation model and evaluate the quantum kernels by running on a classical computer to simulate quantum walk.

5. Methodologies: Topological kernels vs. Entropy kernels

Graph and sub-graph matching is the key procedure for all the kernels. For most of the existing kernels, match mapping is computed via the topological analysis. In this kernel group, graph isomorphism or sub-graph isomorphism is the main method. However, the pairwise matching will cost a lot of time for the kernel computation. Therefore, adding some toy pattern constraints (e.g., edge, fixed-length walk, triangles) or constructing the product graph are the common methods to locate the matching. The product graph is usually an auxiliary structure to locate common sub-graphs, which is constructed by two graphs [22]. However, the product graph will be large if the two graphs are big, which may still lead to unacceptable complexity.

In information theory, mutual information represents the diffusion of two probability distributions. Utilizing the mutual information entropy of the probability distributions of substructures is a novel trend to search the similar substructures. Therefore these methods are called entropy kernels in this paper.

Here 15 popular graph kernels are chosen and shown in Table 1. Every kernel can be grouped according to the above five dimensions. From the groups of these kernels, we find that:

- Most of these kernels are R-convolution, unaligned and local-pattern kernels.
- All the entropy kernels here utilize quantum walk model to compute the probability distribution.
- All of the information theory kernels here belong to the group of entropy kernels. Meanwhile, some R-convolution kernels which detect the similar substructure via the computation of information entropy also belong to this group.

## 3. Complexity Analysis

The computation of graph kernel is erogic and complex. As the increase of graph size and dataset size, the computational cost will increase. In this section, we will make a qualitative comparison on the runtime cost of the 15 chosen graph kernels through the analysis of time complexity.

Table 2 shows the time complexities of all the 15 mentioned kernels. Suppose that the base dataset has K graphs and each graph has N nodes (unattributed nodes) and E edges (undirected and unweight edges). All the complexities are denoted by the three parameters. 

## 4. Quantitative Evaluation

In this section, plenty of graph datasets are utilized to perform a quantitative evaluation on graph kernels in terms of many criteria such as classification accuracy, runtime cost, scalability and applicability. From the aforementioned kernel methods, several graph kernels are evaluated using the following tests.

### 4.1. Datasets

Both real-world datasets and synthetic datasets are used in this paper to evaluate these graph kernels:

**The real-world datasets**. According to the main scope of graph classification, 31 graph datasets from the real world are chosen, including 20 chemical datasets, five image datasets, two social network datasets, three hand-writing datasets and one fingerprint dataset. Among the 31 datasets, some of them are multi-class, such as COIL-DEL and so on. Others are binary class datasets (given in Table A1 in Appendix A). For each object in these datasets, its topological structure is extracted as an unattributed graph, and we try to find the relationship between the natural characteristic and the graph structure. All the datasets can be downloaded from the benchmark website [39].

**The synthetic datasets**. In order to further evaluate the scalability and the applicability of these kernels, some synthetic datasets are chosen or generated, including random graphs, cospectral graphs, regular graphs and strong regular graphs.

Table 3 lists the random graph datasets used for scalability evaluation. According to different node and edge levels, 100 sample graphs are randomly generated with the same size for each class in these datasets. To test the set-based scalability, a dataset is generated which consists of different numbers of random graphs with the same graph size.

Table 4 collects three kinds of similar graphs. CosGraph includes 5048 pairs of 10-node graphs. Each pair of graphs has the same graph spectrum, and is called a cospectral graph pair. RegGraph and SRGraph consist of 31 classes of regular graphs and 11 classes of strong regular graphs respectively. Within one class, each graph is regular or strong regular but not isomorphic with others.

All the synthetic datasets can be downloaded from our github website [40].

### 4.2. Evaluation Criteria

In this paper, our evaluation of graph kernels will mainly focus on several criteria as follows:

1. **Accuracy**. Accuracy is the most important criterion for classification to compare the graph kernels. In this paper, C-SVM [41] is utilized to do the 10-fold cross validation test. In particular, for all the kernels, 10-fold division is the same for every single comparison, and 100 random tests are repeated. Here we use the average probability of the correct-labelled test samples as the accuracy result. Meanwhile, F1 score (macro F1) is used to compute the classification ability for the multi-class problems.
2. **Runtime**. This criterion mainly focuses on the computational cost of a graph kernel for a graph dataset. Because the training procedure belongs to the post-process, we only consider the runtime cost of the computation of the kernel matrix.
3. **Scalability**. To evaluate the runtime cost clearly, scalability is further used to unveil the behavior of the computational time with the increasing number of the graph sizes or graphs in the dataset.
4. **Applicability**. Theoretically, a complete graph kernel is fit for the general graph family, i.e., if graph $G_{A}$ is not isomorphic to graph $G_{B}$, then $k\left(\;\cdot \;,G_{B}\right)\neq k\left(\;\cdot \;,G_{A}\right)$. However, inexact graph kernels may fail to distinguish some similar graphs,, especially the cases of cospectral graphs and regular graphs. We utilize the failure rate as the applicability measurement for the graph kernels.

All the experiments were tested in Matlab R2010b on an Intel Xeon Core E5-1620 CPU with 8 GB memory. All the runtime consumption tests are executed with a single thread.

### 4.3. Results

All the experimental results are shown and analyzed in this subsection.

#### 4.3.1. Accuracy Results

**Figure 2:**
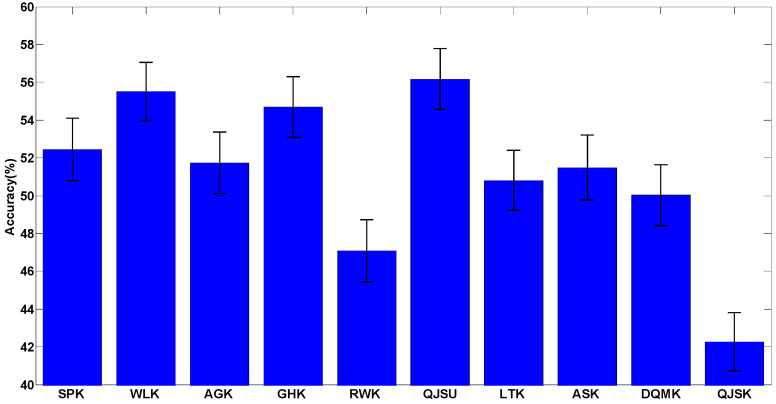
The classification accuracies of the 10 mentioned kernels for the real-world datasets. The detailed information about this test is shown in Table A2.

For all the tests on real-world datasets, a single label is given for the graph under test to predict which class the graph belongs to. It is considered to be correctly classified when the label for the graph under test equals to its true label. The average classification accuracy results are shown in Figure 2, where the real-world datasets used in the tests are given in Appendix A and the accuracy data is given in Table A2 in Appendix B.

**Figure 3:**
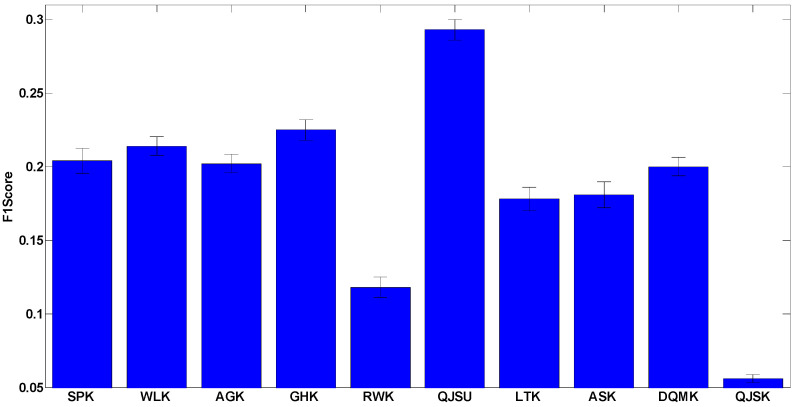
The F1 score of the 10 chosen kernels for the multi-class datasets. The detailed result of this test is shown in Table A3.

Considering the multi-class cases, macro F1 is used as another criterion to evaluate the accuracy performance, which is the harmonic average result of the average precision and the average recall of all the classes in the datasets. Figure 3 illustrates the macro F1 results. The datasets are the multi-class cases in Appendix A, and the macro F1 results (in percentage) are given in Table A3 in Appendix B.

The results show that most of the kernels only achieve over-50% accuracy and over-0.2 F1 score. The reasons are threefold: (1) there are many multi-classes datasets such as COIL (100 classes) and MSRC (20 classes), which are very difficult to correctly recognize; (2) unattributed graphs contain limited information about real-world object, because they neglect the attributes of nodes and edges; (3) compared to the novel multi-kernel method [42], it is more difficult for the single kernel method to capture plenty of complex features of objects.

In addition, we apply statistical tests on the accuracy results. Since some of the accuracy results cannot satisfy the normality assumption, a non-parametric test, the Kruskal-Wallis test, is used. After the statistical test, the significance is 0.631 (the confidence level is 95%), which means that there is no significant difference between the accuracy results of using different graph kernels. This result is obvious since we admit that there is no best kernel for all the datasets, and precisely for this reason, researchers have to design specific graph kernels for a specific given problem. Therefore, a comprehensive kernel evaluation framework is quite useful to kernel comparison. 

Although not significant, there is still a slight advantage of the kernels QJSU, WLK and GHK (which may be due to chance). It can be inferred that some distinguishing ability could be achieved through quantum interference or sub-tree matching. The advantages may be more obvious if some particular classification cases are considered. Therefore, for specific cases, the evaluation process should be reproduced and it is suggested that statistical tests be performed to fully analyze the performance of the kernels.

#### 4.3.2. Runtime Results

Table 5 shows the runtime cost of the 10 chosen kernels for real-world datasets. For each kernel, the runtime of all the 31 real-world datasets are computed. The maximal runtime, minimal runtime and average runtime are listed in lines. Compared with the analyses in Table 2, the runtime evaluation is approximately consistent with the theoretical time complexity comparison.

Generally, SPK is the fastest method because of the fast computation of the shortest path. Meanwhile, WLK and AGK show outstanding runtime performance as well. However, the quantum kernels consume more cost than the classical ones because the simulation of quantum walk costs much runtime on a classical computer. Especially for the case of using DTQW, the runtime cost is nearly the square of that of using RWK, which may be owing to the computation of the evolution of the quantum state.

The Kruskal-Wallis test is applied to the runtime results. When taking 95% degree of confidence, the significance is 0, which means that there are significant differences between the runtime results when using different graph kernels.

#### 4.3.3. Scalability Results

To further explore the scalability, we generate some random graph sets to test the runtime trend with the increasing of the number of node, edge and set size.

• Node-based Scalability

**Figure 4:**
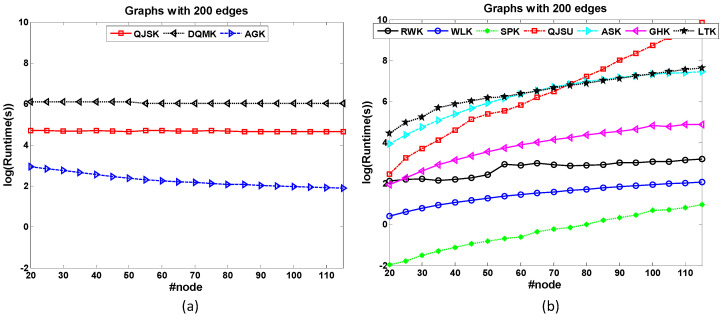
The runtime comparison of kernels for node-based scalability test. (**a**) The runtime costs of QJSK, DQMK and AGK maintain when the graph size increases. (**b**) the ascending runtime trends of other kernels.

The dataset 200-Edge is designed to test the node-based scalability of graph kernels. All the graphs in the dataset have 200 edges. Therefore, the graph density descends as the graph size increases. Figure 4 shows the runtime comparison of kernels for node-based scalability test. Note that the size range of most of the concerned graphs is not large. The x-axis is not changed by the orders of magnitude. In Figure 4a, QJSK, DQMK and AGK have the best scalabilities because the runtime costs of these kernels nearly maintain the same when the graph size increases. These kernels mainly focus on the local patterns which are relative to graph edges. QJSK utilizes DTQW which is quantum walk among edges. Therefore, when the edge number maintains, these kernels are nearly unaffected.

However, other kernels in Figure 4b show bad scalabilities, especially the QJSU which has the sharpest ascending trend as the graph size increases. The main reason is that CTQW is a costly procedure.

• Edge-based Scalability

The dataset 50-Node is generated to test the edge-based scalability of graph kernels. All the graphs have 50 nodes. Unlike the dataset 200-Edge, the graph density increases as the edge number of graphs increases.

**Figure 5:**
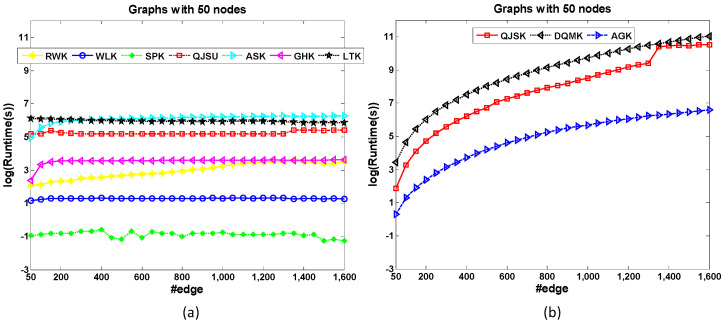
The runtime comparison of kernels for edge-based scalability test. Compared with QJSK, DQMK and AGK shown in (**b**), the kernels in (**a**) almost maintain the runtime cost when the graph density increases.

Figure 5 shows the runtime comparison of kernels for edge-based scalability test. Most kernels show good scalabilities when graph density increases (see Figure 5a), except AGK, QJSK and DQMK in Figure 5b. RWK locates the walk pattern of the graphs therefore it is sensible to the graph density. QJSK and DQMK utilize the edge-based discrete-time quantum walk to compute the similarity, which is a high complexity operation when the graph is dense. Therefore, the runtime costs of these 3 kernels increase significantly when the edges increase (see Figure 5b). It is found that this observation is nearly opposite with the result of the above Node-based Scalability experiment.

• Set-based Scalability

**Figure 6:**
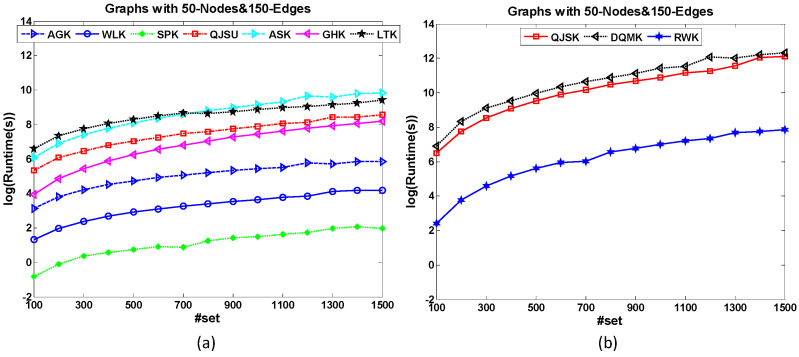
The runtime comparison of kernels for set-based scalability test. Compared with QJSK, DQMK and RWK shown in (**b**), the kernels in (**a**) show slower increasing trends when the graph set increases.

The dataset 50-Node&150-Edge is designed to test the set-based scalability of graph kernels. All the graph samples have the same amount of nodes and edges. Figure 6 shows the runtime comparison of kernels for set-based scalability test. Based on the formal definition of the kernel, the kernel matrix used in the kernel-based classification is pairwise and the matrix size relates to the graph number of the dataset. Therefore, all the kernels will cost more runtime when the graph set increases. Compared with other methods, the increasing trends of the runtime costs of QJSK, DQMK and RWK are more significant. For a large dataset, these three kernels cannot work well within an acceptable time.

• Normalized Evaluation

For every kernel, the runtime cost is related with many factors as listed in Table 2. The factor set is assumed to be {x,y,…z}. Take factor x as an example. In order to make a normalized standard evaluation on the x-based scalability of a kernel method, we fix all the other factors in the dataset and use the following function to compute the normalized scalability:

(6) $$Scalability_{x}=\frac{1}{n-1}\sum_{i=1}^{n-1}\frac{\ln T_{x_{i},y\ldots z}-\ln T_{x_{i+1},y\ldots z}}{\ln x_{i}-\ln x_{i+1}}$$

where $T_{x,y\ldots z}$ denotes the runtime cost for a dataset with the relative factors {x,y,…z}.

Actually, the x-based scalability should be evaluated by the derivatives of the sub-function $\frac{\partial T_{x}}{\partial x}$. The higher the value is, the x-based scalability is worse. However, for an arbitrary kernel, the sub-cost $T_{x}$ is difficult to test. Approximately, we could assume that every factor is independent with each other and the x-based asymptotic complexity is about $O\left(x^{k}\right)$. It can be easily proved that Equation (6) can be used to compute $\frac{\partial T_{x}}{\partial x}$ approximately.

Note that even if the scalability equals to 1, it does not mean that the runtime cost changes linearly with the factor. This function should be considered as an approximate method to measure the scalability quantitatively.

The node-based, edge-based and set-based normalized scalabilities of the 10 graph kernels are given in Table 6.

#### 4.3.4. Applicability Results

Some similar and non-isomorphic graphs are usually difficult to distinguish via inexact graph comparison methods. Therefore, a graph kernel cannot be applied to some kinds of graphs. In this subsection, the distinguishing ability for similar graphs is used to compare the applicability of these graph kernels. Here similar graphs are the graph pairs or graph groups with similar structure.

Table 7 shows the failure rates of these graph kernels for distinguishing the similar graph pairs collected in Table 4, including the cospectral graphs, regular graphs and strong regular graphs.

The Kruskal-Wallis test under the 95% degree of confidence is conducted. The significance is 0.014. Therefore, the graph kernels have significant influence on the applicability. RWK is the worst kernel, which cannot be used to distinguish these similar graphs. WLK could only locate the difference of the cospectral graphs, but fails for regular graphs. On the contrary, DQMK, LTK, QJSU and AGK achieve the best distinguishing abilities, even for the strong regular graphs.

Generally, the quantum kernels show better applicability. Because the slight topological difference will be amplified by quantum interference, and thus better distinguishing ability is achieved. Therefore, when the sample graphs are similar and difficult to be classified, quantum kernels will be better choices.

## 5. Discussion

According to the evaluations in Section 4, seven criteria are considered for each kernel including classification accuracy, F1Score, runtime cost, node-based scalability, edge-based scalability, set-based scalability and distinguishing ability. The normalized ability value (using the ability *X* of kernel *K* as an example) is defined as follows:

(7) $$Ability_{X}=\frac{\left|X_{K}-X_{worst}\right|}{\left|X_{best}-X_{worst}\right|}$$

where $X_{best}$ and $X_{worst}$ are the ability value of the optimal kernel and the worst kernel in all the 10 mentioned kernels respectively.

According to Section 2.3, all the graph kernels can be grouped according to five different dimensions. Here we focus on the comparison of the graph kernel groups to explore the advantages and disadvantages of all the kernel groups. For each kernel group, we compare the average abilities of all the kernels in this group.

**Figure 7:**
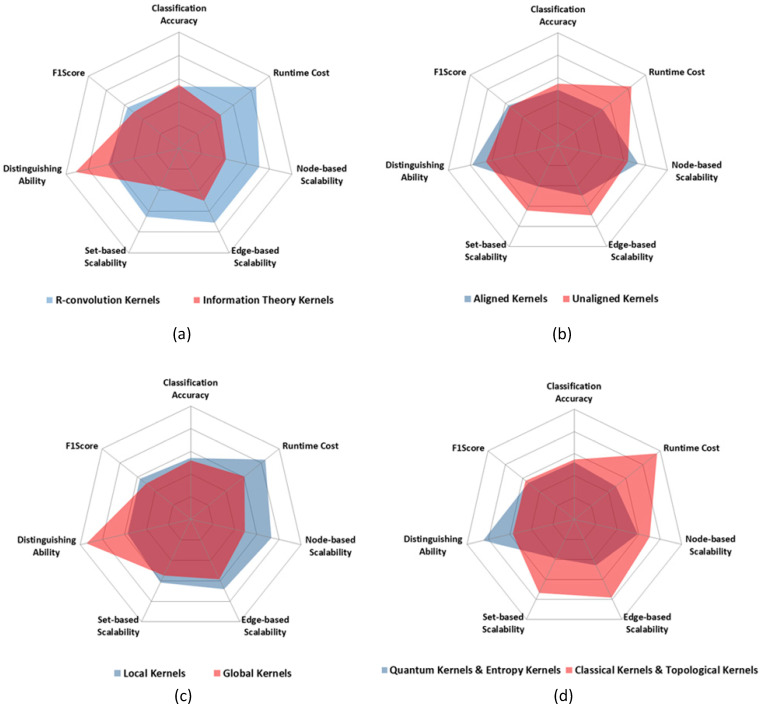
The ability comparison of all the 5 graph kernel groups in 7 criteria. (**a**) R-convolution kernels vs. Information theory kernels. (**b**) Aligned kernels vs. Unaligned kernels. (**c**) Local kernels vs. Global kernels. (**d**) Quantum kernels vs. Classical kernels. Note that for the 10 chosen kernels, the radar figure of Entropy kernels vs. Topological kernels is the same with that of Quantum kernels vs. Classical kernels.

Figure 7 shows the comparison results of the five graph kernel groups. In the radar figures in Figure 7, the bigger the ability scope is, the better the graph kernel group is. And for each criteria of the comparison, the bigger the value is, the stronger the ability is.

Through the statistical analyses, we find out that:

- R-convolution kernels perform better on scalabilities and runtime cost, while the information theory kernels show better abilities on accuracy and applicability. The information theory kernels utilize the global probability distribution diffusion of two graphs to measure the graph similarity. Therefore, compared with local pattern matching of R-convolution kernels, the information theory kernels result in the better accuracy and applicability.
- Aligned kernels have stronger applicability and node-based scalability but are weaker than unaligned kernels on the other criteria. Through graph alignment, the vertex mapping characteristic is found out before kernel computation. Meanwhile, the slight difference of the similar graph pairs can also be located in the alignment procedure. Therefore, after graph alignment, the kernel methods can utilize the vertex mapping directly, which leads to a well node-based scalability.
- Global kernels, quantum kernels and entropy kernels are worse than their counterpart kernel group in all the other criteria except the distinguishing ability (applicability). It unveils that if good applicability is needed, more complex computations are needed in the kernel method, such as the above kinds of graph kernels.

Above all, R-convolution, unaligned, local-pattern, classical and topological kernels have better ability scope and show more advantages. However, these kinds of kernels lack powerful applicability. The reasons are twofold. Firstly, a complete graph kernel can distinguish all the non-isomorphic graph pairs, which means it has the best applicability. However, it is NP-hard. Therefore, to achieve a powerful applicability, the computation cost will be great. Secondly, distinguishing slight differences will lead to bad generalization performance, and thus result in low accuracy.

## 6. Conclusions

In this paper, a comprehensive evaluation of graph kernels for unattributed graphs is introduced. According to five different dimensions of the design details, all the existing graph kernels can be catalogued and 10 representative graph kernels are chosen to be completed compared using plenty of real-world and synthetic datasets. For each kernel, we focus on seven criteria to evaluate the performance of the kernel, namely, the classification accuracy, runtime cost, node-based scalability, edge-based scalability, set-based scalability and applicability. Through the kernel group comparison, it is found that the R-convolution, unaligned, local-pattern, classical and topological kernels achieve better performance in all the criteria except for the applicability.

Ten chosen graph kernels may not be enough to represent all the existing graph kernel methods. Therefore, some conclusions in this paper should be seen as guidelines which are useful for choosing an optimal kernel for graph classification or designing a novel kernel. As to the future work, more kernels will be included and graph kernels for attributed graphs will be considered.

## Figures and Tables

**Table 1:** The 15 groups of chosen graph kernels.

Kernel Name	Framework	Aligned	Matching Pattern	Computing Model	Methodology
**SPK [23]**	R-convolution	No	Local (Path)	Classical	Topology
**WLK [9]**	R-convolution	No	Local (Subtree)	Classical	Topology
**AGK [10]**	R-convolution	No	Local (Graphlet)	Classical	Topology
**GHK [36]**	R-convolution	No	Local (Path)	Classical	Topology
**RWK [22]**	R-convolution	No	Local (Walk)	Classical	Topology
**QJSU [11]**	Information Theory	No	Global	CTQW	Entropy
**LTK [32]**	R-convolution	No	Global	Classical	Topology
**ASK [21]**	R-convolution	Yes	Local (Subtree)	CTQW	Entropy
**DQMK [35]**	R-convolution	Yes	Local (Edge)	DTQW	Entropy
**QJSK [12]**	Information Theory	No	Global	DTQW	Entropy
**B** **WK [6]**	R-convolution	No	Local(Walk)	Classical	Topology
**JTK [25]**	Information Theory	No	Global	CTQW	Entropy
**NSPDK [37]**	R-convolution	No	Local(Subgraph)	Classical	Topology
**CPK [38]**	R-convolution	No	Local(Cycle)	Classical	Topology
**MLGK [33]**	Information Theory	No	Mix	Classical	Topology

**Table 2:** The time complexities of 15 chosen graph kernels.

Kernel Name	Complexity	P.S.
**SPK**	$O\left(KN^{4}+K^{3}\right)$	-
**WLK**	$O\left(KN^{2}+K^{2}N^{2}\right)$	
**AGK**	$O\left(KN^{4}+K^{3}\right)$	e.g., graphlet size is 4
**GHK**	$O\left(K^{2}N^{2}\left(E+\log N+N^{2}\right)\right)$	
**RWK**	$O\left(K^{2}N^{3}\right)$	-
**QJSU**	$O\left(K^{2}N^{3}\right)$	-
**LTK**	$O\left(K^{2}\left(EN^{2}+N^{3}\right)\right)$	Gaussian Kernel as the base kernel
**ASK**	$O\left(K^{2}N^{4}\right)$	
**DQMK**	$O\left(K^{2}NE^{3}\right)$	
**QJSK**	$O\left(K^{2}NE^{3}\right)$	
**BWK**	$O\left(K^{2}N^{3}\right)$	
**JTK**	$O\left(K^{2}N^{2}+KN^{3}\right)$	
**NSPDK**	$O\left(K^{2}N^{2}E\log E\right)$	
**CPK**	$O\left(K\left(N^{2}+E\right)\right)$	
**MLGK**	$O\left(K^{2}N^{5}\right)$	

**Table 3:** The random graph datasets for scalability evaluation.

Dataset Name	Statistics			
	#Set	#Nodes	#Edges	#Class
**50-Node**	3000	50	50:50:1500	30
**200-Edge**	2000	20:5:115	200	20
**50-Node&150-Edge**	100:100:1500	50	150	15

**Table 4:** Three similar-graph datasets. In CosGraph, every cospectral graph pair is used as a test. In RegGraph and SRGraph, paired compassions of the graphs in each class are done using kernel methods.

Dataset Name	Statistics			
	#set	#Class	Avg. #Nodes	#Test Pairs
**CosGraph**	10,096	5048	10	5048
**RegGraph**	6490	31	16.34	885,128
**SRGraph**	7303	11	37.63	5,099,490

**Table 5:** The runtime cost of graph kernels for real-world datasets.

Kernel Name	Minimum Runtime	Maximum Runtime	Average Runtime
**SPK**	0.19 s	11.81 s	2.97 s
**WLK**	2.42 s	2.01 m	26 s
**AGK**	3.09 s	6.38 m	1.50 m
**GHK**	14.02 s	4.42 h	27.93 m
**RWK**	22.40 s	4.27 h	45.6 m
**QJSU**	18.02 s	3.95 h	50.3 m
**LTK**	67.99 s	4.86 h	1.05 h
**ASK**	84.82 s	14.71 h	2.55 h
**DQMK**	85.50 s	47.17 h	7.85 h
**QJSK**	62.54 s	70.44 h	10.6 h

**Table 6:** The three kinds of normalized scalabilities of the 10 mentioned kernels. The results in red italic font denote bad scalabilities we observe in Figure 4, Figure 5 and Figure 6, while the results in blue bold font are outstanding scalabilities.

Kernel Name	Node-Based Scalability	Edge-Based Scalability	Set-Based Scalability
**SPK**	1.96	**−0.41**	1.04
**WLK**	0.96	**−0.02**	1.14
**AGK**	**−0.58**	*1.96*	**0.98**
**GHK**	1.60	0.09	1.69
**RWK**	0.68	0.61	*2.06*
**QJSU**	*5.00*	0.17	1.34
**LTK**	1.82	**−0.12**	1.10
**ASK**	1.86	0.22	1.48
**DQMK**	**−0.04**	*2.48*	*2.07*
**QJSK**	**−0.04**	*3.08*	*2.32*

**Table 7:** The failure rates of all the kernels for distinguishing the similar graphs.

Kernel Name	Cospectral Graphs	Regular Graphs	Strong Regular Graphs	Average
**SPK**	33.16%	1.14%	100%	44.77%
**WLK**	1.66%	100%	100%	67.22%
**AGK**	5.96%	4.87%	3.82%	4.88%
**GHK**	18.82%	0.12%	100%	39.65%
**RWK**	100%	100%	100%	100%
**QJSU**	0%	2.79%	0.65%	1.15%
**L** **TK**	0%	0%	0%	0%
**ASK**	33.16%	1.14%	95.96%	43.42%
**DQMK**	0%	0%	0%	0%
**QJSK**	33.16%	1.14%	13.60%	15.97%

## Appendix A. The Real-World Datasets

All the real-world datasets are listed in Table A1.

**Table A1:** The detailed information of the real-world datasets. The dataset density is calculated by the average edges divided by the average nodes.

Dataset Name	Statistics					Description
	#Set	#Class	Avg. #Nodes	Avg. #Edges	Density	
**Mutagenicity**	4337	2	30.32	30.77	1.01	Chemical Molecule
**PTC_MM**	336	2	13.97	14.32	1.03	Chemical Molecule
**PTC_FM**	349	2	14.11	14.48	1.03	Chemical Molecule
**PTC_MR**	344	2	14.29	14.69	1.03	Chemical Molecule
**PTC_FR**	351	2	14.56	15	1.03	Chemical Molecule
**AIDS**	2000	2	15.69	16.2	1.03	Chemical Molecule
**DHFR**	467	2	42.43	44.54	1.05	Chemical Molecule
**COX2**	467	2	41.22	43.45	1.05	Chemical Molecule
**FRANKENSTEIN**	4337	2	16.9	17.88	1.06	Chemical Molecule
**BZR**	405	2	35.75	38.36	1.07	Chemical Molecule
**NCI1**	4110	2	29.87	32.3	1.08	Chemical Molecule
**NCI109**	4127	2	29.68	32.13	1.08	Chemical Molecule
**MUTAG**	188	2	17.93	19.79	1.10	Chemical Molecule
**PROTEINS**	1113	2	39.06	72.82	1.86	Chemical Molecule
**PROTEINS_full**	1113	2	39.06	72.82	1.86	Chemical Molecule
**ENZYMES**	600	6	32.63	62.14	1.90	Chemical Molecule
**BZR_MD**	306	2	21.3	225.06	10.57	Chemical Molecule
**ER_MD**	446	2	21.33	234.85	11.01	Chemical Molecule
**DHFR_MD**	393	2	23.87	283.01	11.86	Chemical Molecule
**COX2_MD**	303	2	26.28	335.12	12.75	Chemical Molecule
**COIL-RAG**	3900	100	3.01	3.02	1.00	Image
**MSRC_21C**	209	20	40.28	96.6	2.40	Image
**MSRC_9**	221	8	40.58	97.94	2.41	Image
**COIL-DEL**	3900	100	21.54	54.24	2.52	Image
**MSRC_21**	563	20	77.52	198.32	2.56	Image
**Letter-low**	2250	15	4.68	3.13	0.67	Handwriting
**Letter-high**	2250	15	4.67	4.5	0.96	Handwriting
**Letter-med**	2250	15	4.67	4.5	0.96	Handwriting
**IMDB-BINARY**	1000	2	19.77	96.53	4.88	Social Network
**IMDB-MULTI**	1500	3	13	65.94	5.07	Social Network
**Fingerprint**	2800	4	5.42	4.42	0.82	Fingerprint

## Appendix B. The Complete Results of Accuracy Test

The detailed results of the accuracy of the classification test are shown in Table A2 and Table A3.

**Table A2:** The complete results of the classification accuracy. Every item is comprised of the average accuracy (%) and the standard deviation of 100 repeated tests. The bold numbers are the best result among the ten kernels for each dataset.

Datasets	SPK	WLK	AGK	GHK	RWK	QJSU	LTK	ASK	DQMK	QJSK
**AIDS**	99.32 ± 0.59	98.75 ± 0.87	98.78 ± 0.84	99.33 ± 0.58	79.99 ± 2.67	**99.73 ± 0.34**	99.54 ± 0.46	96.79 ± 1.24	79.99 ± 2.67	79.99 ± 2.67
**BZR**	79.95 ± 2.31	**87.75 ± 1.53**	78.96 ± 2.35	83.82 ± 1.86	78.61 ± 2.34	83.26 ± 2.01	79.23 ± 2.26	79.29 ± 2.56	78.61 ± 2.34	78.61 ± 2.34
**BZR_MD**	60.71 ± 2.51	59.25 ± 2.82	48.96 ± 0.98	60.56 ± 2.36	61.49 ± 1.26	**62.33 ± 2.00**	60.59 ± 2.41	60.87 ± 2.26	59.08 ± 1.15	55.41 ± 2.36
**COIL-DEL**	12.04 ± 1.60	12.43 ± 1.51	8.80 ± 1.41	**18.11 ± 1.75**	0.94 ± 0.49	7.83 ± 1.23	7.70 ± 1.16	4.46 ± 1.05	6.01 ± 1.10	0.84 ± 0.08
**COIL-RAG**	4.99 ± 1.11	5.89 ± 1.14	3.34 ± 0.87	5.77 ± 1.18	0.83 ± 0.05	**6.16 ± 1.13**	5.20 ± 1.16	2.45 ± 0.76	3.76 ± 0.93	0.83 ± 0.05
**COX2**	78.15 ± 5.64	**78.87 ± 5.63**	78.15 ± 5.64	78.65 ± 5.74	78.15 ± 5.64	78.69 ± 5.94	78.17 ± 5.57	78.15 ± 5.64	78.15 ± 5.64	78.08 ± 5.69
**COX2_MD**	47.95 ± 1.12	47.51 ± 1.86	47.95 ± 1.12	**48.00 ± 1.14**	47.00 ± 1.83	46.80 ± 2.00	47.57 ± 1.19	47.44 ± 1.08	46.77 ± 1.33	47.85 ± 1.01
**DHFR**	70.09 ± 5.89	**82.13 ± 4.31**	61.23 ± 5.48	79.77 ± 4.75	61.23 ± 5.48	79.03 ± 4.09	60.91 ± 5.04	47.40 ± 4.80	76.77 ± 4.96	61.23 ± 5.48
**DHFR_MD**	**68.11 ± 3.61**	67.07 ± 3.57	**68.11 ± 3.61**	66.81 ± 3.61	67.55 ± 3.57	66.88 ± 3.62	67.12 ± 3.66	67.72 ± 3.68	**68.11 ± 3.61**	**68.11 ± 3.61**
**ER_MD**	59.65 ± 3.50	63.14 ± 3.62	59.14 ± 3.07	62.62 ± 3.26	62.50 ± 3.03	61.65 ± 3.31	62.78 ± 3.01	**63.19 ± 3.62**	59.04 ± 3.05	59.14 ± 3.07
**ENZYMES**	28.70 ± 5.58	**37.39 ± 6.48**	26.65 ± 5.78	37.34 ± 6.58	11.63 ± 3.12	31.97 ± 6.21	22.89 ± 5.19	30.27 ± 5.67	28.91 ± 5.71	19.56 ± 2.44
**Fingerprint**	26.62 ± 2.81	27.23 ± 2.93	29.75 ± 3.04	26.68 ± 2.69	24.57 ± 2.75	30.16 ± 3.12	29.56 ± 3.21	24.74 ± 2.62	**30.73 ± 3.00**	24.14 ± 2.85
**FRANKENSTEIN**	60.46 ± 2.35	**72.40 ± 1.89**	59.46 ± 2.29	67.33 ± 2.08	57.54 ± 2.67	66.91 ± 2.22	62.07 ± 2.25	63.33 ± 2.00	63.77 ± 2.39	52.51 ± 3.16
**IMDB-BINARY**	59.09 ± 5.21	**72.45 ± 4.33**	64.92 ± 5.22	71.74 ± 4.47	67.50 ± 5.14	62.10 ± 5.24	61.75 ± 5.21	63.57 ± 5.03	46.40 ± 4.30	50.36 ± 5.57
**IMDB-MULTI**	40.71 ± 4.63	**50.96 ± 4.37**	40.11 ± 4.25	50.45 ± 3.61	46.20 ± 4.52	43.24 ± 4.14	45.81 ± 3.72	42.81 ± 5.15	49.02 ± 4.93	43.51 ± 4.15
**Letter-high**	28.99 ± 3.02	33.87 ± 2.84	32.03 ± 3.09	34.20 ± 3.03	13.25 ± 2.23	**46.36 ± 3.13**	30.71 ± 3.05	28.20 ± 3.08	32.89 ± 3.50	26.90 ± 3.03
**Letter-low**	32.82 ± 2.87	39.30 ± 3.18	43.92 ± 3.11	37.15 ± 3.31	6.86 ± 2.70	**78.90 ± 2.50**	33.26 ± 3.03	33.03 ± 5.61	46.74 ± 3.29	27.71 ± 2.09
**Letter-med**	30.63 ± 2.73	36.36 ± 3.40	39.70 ± 3.15	35.62 ± 3.27	7.16 ± 2.64	**74.02 ± 2.87**	30.82 ± 3.16	28.66 ± 5.28	43.72 ± 3.25	27.10 ± 2.17
**Mutagenicity**	63.64 ± 2.18	**78.48 ± 1.86**	59.62 ± 2.38	69.34 ± 2.11	55.32 ± 2.39	69.07 ± 2.10	60.17 ± 2.17	62.44 ± 2.04	58.71 ± 2.67	55.32 ± 2.39
**MSRC_9**	16.53 ± 1.53	13.00 ± 2.98	8.05 ± 1.10	**19.29 ± 2.70**	8.49 ± 1.28	15.00 ± 0.37	9.82 ± 1.61	17.78 ± 3.10	14.73 ± 2.02	9.38 ± 2.47
**MSRC_21**	**12.86 ± 0.68**	7.52 ± 0.77	5.66 ± 1.74	11.35 ± 1.83	4.12 ± 0.49	6.92 ± 1.18	5.02 ± 1.87	7.12 ± 1.22	3.54 ± 1.22	4.39 ± 2.10
**MSRC_21C**	**19.14 ± 2.90**	11.82 ± 1.40	13.24 ± 1.69	14.32 ± 2.44	12.26 ± 1.14	17.12 ± 1.35	15.04 ± 1.41	15.52 ± 1.25	11.28 ± 1.22	11.78 ± 1.27
**MUTAG**	82.96 ± 3.65	81.69 ± 2.31	80.84 ± 3.52	**85.40 ± 2.65**	77.00 ± 3.41	82.67 ± 2.19	83.29 ± 2.81	84.20 ± 3.46	76.42 ± 2.88	79.00 ± 3.41
**NCI1**	61.76 ± 2.40	**81.77 ± 1.79**	62.48 ± 2.29	68.02 ± 2.36	57.95 ± 1.46	66.25 ± 2.21	62.84 ± 2.47	64.49 ± 2.55	65.18 ± 2.33	57.82 ± 1.60
**NCI109**	62.16 ± 2.33	**82.29 ± 1.89**	62.41 ± 2.28	67.34 ± 2.51	59.80 ± 2.11	66.10 ± 2.56	62.58 ± 2.44	63.22 ± 2.56	64.88 ± 2.25	59.62 ± 2.18
**PTC_FM**	59.09 ± 2.23	58.64 ± 2.36	60.08 ± 2.20	59.62 ± 2.11	58.75 ± 2.89	59.91 ± 2.15	**61.22 ± 2.06**	59.39 ± 2.52	59.33 ± 1.76	58.39 ± 2.00
**PTC_FR**	65.43 ± 3.58	65.30 ± 4.13	65.50 ± 3.64	65.31 ± 3.62	**65.66 ± 3.56**	64.49 ± 3.62	65.51 ± 3.35	65.10 ± 3.27	65.62 ± 3.57	65.49 ± 3.57
**PTC_MM**	60.92 ± 2.69	61.98 ± 2.01	**62.05 ± 2.58**	61.45 ± 2.59	61.27 ± 2.64	59.37 ± 2.48	59.60 ± 2.63	60.99 ± 2.49	61.09 ± 2.73	61.08 ± 2.76
**PTC_MR**	56.39 ± 3.51	56.68 ± 3.69	56.35 ± 4.29	55.94 ± 4.34	56.05 ± 3.13	56.29 ± 4.17	56.92 ± 4.14	56.22 ± 4.88	**57.29 ± 4.20**	55.61 ± 4.23
**PROTEINS**	72.50 ± 4.38	72.77 ± 4.11	71.29 ± 4.26	**74.13 ± 3.97**	70.13 ± 4.88	71.79 ± 4.03	71.29 ± 4.15	72.00 ± 4.32	66.05 ± 4.61	70.13 ± 4.88
**PROTEINS_full**	72.36 ± 3.86	72.56 ± 4.19	70.88 ± 3.95	**73.81 ± 3.78**	69.26 ± 4.31	71.62 ± 4.26	70.88 ± 3.96	71.74 ± 4.01	65.25 ± 4.47	69.26 ± 4.31
**Average**	51.44 ± 3.00	55.39 ± 2.90	50.59 ± 2.94	54.49 ± 2.98	46.10 ± 2.77	**55.90 ± 2.83**	50.64 ± 2.90	51.28 ± 3.19	50.58 ± 3.00	47.07 ± 2.87

**Table A3:** The complete result of the F1 score test. Every item shows the average score (%) and the standard deviation of 100 repeated tests. The bold numbers are the best result among the ten kernels for each dataset.

Datasets	SPK	WLK	AGK	GHK	RWK	QJSU	LTK	ASK	DQMK	QJSK
**COIL-DEL**	12.55 ± 1.39	13.07 ± 1.59	10.98 ± 1.6	**17.59 ± 2.21**	3.23 ± 0.72	11.77 ± 1.67	5.01 ± 0.71	4.74 ± 0.69	6.67 ± 1.74	1.02 ± 0.1
**COIL-RAG**	2.91 ± 0.79	4.31 ± 0.72	2.3 ± 0.74	4.25 ± 1.13	1.78 ± 0.77	**4.63 ± 1.26**	3.77 ± 0.94	2.34 ± 1.14	3.40 ± 0.80	0.83 ± 0.11
**ENZYMES**	30.61 ± 3.38	37.35 ± 4.5	30.04 ± 7.31	**37.67 ± 4.87**	11.76 ± 5.06	33.98 ± 7.34	22.8 ± 6.26	30.7 ± 4.77	26.94 ± 5.85	5.88 ± 0.67
**Fingerprint**	5.78 ± 1.51	6.63 ± 0.54	6.54 ± 1.59	6.26 ± 0.64	4.43 ± 1.01	7.14 ± 1.1	6.37 ± 1.5	3.35 ± 1.13	**7.17 ± 0.78**	2.78 ± 0.34
**IMDB-MULTI**	40.89 ± 3.08	51.67 ± 3.09	42.5 ± 1.61	**51.94 ± 3.16**	48.29 ± 4.23	44.1 ± 4.01	46.55 ± 3.75	46.0 ± 2.57	37.76 ± 3.99	29.86 ± 4.67
**Letter-high**	28.98 ± 3.74	31.69 ± 2.01	31.73 ± 2.02	34.28 ± 2.86	11.08 ± 1.83	**47.86 ± 2.37**	31.53 ± 3.73	27.3 ± 2.45	38.03 ± 2.77	6.09 ± 1.03
**Letter-low**	32.21 ± 3.66	35.84 ± 2.96	36.17 ± 1.96	33.75 ± 1.83	11.91 ± 3.05	**79.32 ± 1.28**	32.25 ± 2.46	31.82 ± 5.79	44.17 ± 1.59	6.72 ± 0.89
**Letter-med**	28.16 ± 2.75	32.95 ± 3.75	33.67 ± 1.85	33.3 ± 2.57	12.87 ± 4.46	**74.1 ± 3.23**	29.49 ± 4.31	26.73 ± 3.13	41.3 ± 1.14	5.00 ± 0.29
**MSRC_9**	**22.13 ± 2.52**	10.23 ± 1.66	18.0 ± 1.85	13.68 ± 2.34	14.28 ± 1.78	9.34 ± 1.12	9.40 ± 2.60	16.47 ± 3.52	12.38 ± 2.56	1.78 ± 0.19
**MSRC_21**	**14.02 ± 2.77**	8.47 ± 1.37	6.41 ± 1.16	10.85 ± 2.53	8.15 ± 1.67	5.88 ± 0.14	3.43 ± 0.91	4.26 ± 1.76	0.49 ± 0.16	0.74 ± 0.10
**MSRC_21C**	**6.56 ± 0.47**	2.96 ± 0.29	3.81 ± 0.43	3.96 ± 0.35	1.97 ± 0.2	3.94 ± 0.43	5.37 ± 0.36	5.28 ± 0.31	2.11 ± 0.14	1.01 ± 0.11
**Average**	20.4 ± 2.7	21.40 ± 2.0	20.2 ± 2.0	22.5 ± 2.2	11.8 ± 2.2	**29.3 ± 2.2**	17.8 ± 2.5	18.10 ± 2.80	20.0 ± 2.00	5.6 ± 0.87

## References

[1] Ta V.T., Lézoray O., Elmoataz A., Schüpp S. (2009). Graph-based tools for microscopic cellular image segmentation. Pattern Recognit..

[2] Raymond J.W., Willett P. (2002). Maximum common subgraph isomorphism algorithms for the matching of chemical structures. J. Comput.-Aided Mol. Des..

[3] Fan W. Graph pattern matching revised for social network analysis. Proceedings of the International Conference on Database Theory.

[4] Ngomo A.C.N., Schumacher F. BorderFlow: A Local Graph Clustering Algorithm for Natural Language Processing. Proceedings of the International Conference on Computational Linguistics and Intelligent Text Processing.

[5] Mahé P., Vert J.P. (2009). Graph kernels based on tree patterns for molecules. Mach. Learn..

[6] Aziz F., Wilson R.C., Hancock E.R. (2013). Backtrackless walks on a graph. IEEE Trans. Neural Netw. Learn. Syst..

[7] Neuhaus M., Bunke H. (2007). Bridging the Gap between Graph Edit Distance and Kernel Machines.

[8] Torresani L., Kolmogorov V., Rother C. Feature correspondence via graph matching: Models and global optimization. Proceedings of the Computer Vision—ECCV.

[9] Shervashidze N., Schweitzer P., Leeuwen E.J., Mehlhorn K., Borgwardt K.M. (2011). Weisfeiler-lehman graph kernels. J. Mach. Learn. Res..

[10] Shervashidze N., Vishwanathan S.V., Petri T., Mehlhorn K., Borgwardt K. Efficient graphlet kernels for large graph comparison. Proceedings of the Twelfth International Conference on Artificial Intelligence and Statistics.

[11] Bai L., Rossi L., Torsello A., Hancock E.R. (2015). A quantum Jensen–Shannon graph kernel for unattributed graphs. Pattern Recognit..

[12] Bai L., Rossi L., Cui L., Zhang Z., Ren P., Bai X., Hancock E. (2017). Quantum kernels for unattributed graphs using discrete-time quantum walks. Pattern Recognit. Lett..

[13] Orsini F., Frasconi P., De Raedt L. Graph invariant kernels. Proceedings of the 24th International Conference on Artificial Intelligence (AAAI 2015).

[14] Morris C., Kriege N.M., Kersting K., Mutzel P. Faster kernels for graphs with continuous attributes via hashing. Proceedings of the IEEE 16th International Conference on Data Mining (ICDM 2016).

[15] Berg J., Karp R.M. (2004). Local graph alignment and motif search in biological networks. Proc. Natl. Acad. Sci. USA.

[16] Ferreira J.C., Menegatto V.A. (2009). Eigenvalues of integral operators defined by smooth positive definite kernels. Integral Equ. Oper. Theory.

[17] Balcan M.F., Blum A. On a theory of learning with similarity functions. Proceedings of the International Conference on Machine Learning.

[18] Chen Y., Gupta M.R., Recht B. Learning kernels from indefinite similarities. Proceedings of the International Conference on Machine Learning.

[19] Haussler D. (1999). Convolution Kernels on Discrete Structures.

[20] Bai L. (2014). Information Theoretic Graph Kernels.

[21] Bai L., Rossi L., Zhang Z., Hancock E. An aligned subtree kernel for weighted graphs. Proceedings of the International Conference on Machine Learning (ICML 2015).

[22] Gärtner T., Flach P., Wrobel S. On graph kernels: Hardness results and efficient alternatives. Proceedings of the Learning Theory and Kernel Machines 16th Annual Conference on Learning Theory and 7th Kernel Workshop (COLT/Kernel 2003).

[23] Borgwardt K.M., Kriegel H.P. Shortest-path kernels on graphs. Proceedings of the 5th IEEE International Conference on Data Mining (ICDM 2005).

[24] Rossi L., Torsello A., Hancock E.R. (2015). Measuring Graph Similarity through Continuous-Time Quantum Walks and the Quantum Jensen-Shannon Divergence. Phys. Rev. E.

[25] Bai L., Rossi L., Bunke H., Hancock E.R. Hancock: Attributed Graph Kernels Using the Jensen-Tsallis q-Differences. Proceedings of the Joint European Conference on Machine Learning and Knowledge Discovery in Databases.

[26] Bai L., Edwin R. (2013). Hancock: Graph Kernels from the Jensen-Shannon Divergence. J. Math. Imaging Vis..

[27] Bai L., Zhang Z., Wang C., Bai X., Hancock E.R. Hancock: A Graph Kernel Based on the Jensen-Shannon Representation Alignment. Proceedings of the IJCAI.

[28] Rossi L., Torsello A., Hancock E.R. (2015). Unfolding Kernel Embeddings of Graphs: Enhancing Class Separation through Manifold Learning. Pattern Recognit..

[29] Fröhlich H., Wegner J.K., Sieker F., Zell A. Optimal assignment kernels for attributed molecular graphs. Proceedings of the 22nd International Conference on Machine Learning (ICML 2005).

[30] Vert J.P. (2008). The optimal assignment kernel is not positive definite. arXiv.

[31] Kriege N.M., Giscard P.L., Wilson R. On valid optimal assignment kernels and applications to graph classification. Proceedings of the Advances in Neural Information Processing Systems (NIPS 2016).

[32] Johansson F., Jethava V., Dubhashi D., Bhattacharyya C. Global graph kernels using geometric embeddings. Proceedings of the 31st International Conference on Machine Learning (ICML 2014).

[33] Kondor R., Pan H. The multiscale Laplacian graph kernel. Proceedings of the Advances in Neural Information Processing Systems (NIPS 2016).

[34] Childs A.M. (2009). Universal computation by quantum walk. Phys. Rev. Lett..

[35] Bai L., Zhang Z., Ren P., Rossi L., Hancock E.R. An edge-based matching kernel through discrete-time quantum walks. Proceedings of the International Conference on Image Analysis and Processing (ICIAP 2015).

[36] Feragen A., Kasenburg N., Petersen J., de Bruijne M., Borgwardt K. Scalable kernels for graphs with continuous attributes. Proceedings of the Advances in Neural Information Processing Systems (NIPS 2013).

[37] Costa F., de Grave K. Fast neighborhood subgraph pairwise distance kernel. Proceedings of the ICML.

[38] Horváth T., Gärtner T., Wrobel S. Cyclic pattern kernels for predictive graph mining. Proceedings of the KDD.

[39] The Graph Kernel Benchmarks. https://ls11-www.cs.tu-dortmund.de/staff/morris/graphkerneldatasets.

[40] The Datasets and the Matlab Codes. https://github.com/YiZhangNUDT/graph_kernel_test.

[41] Chang C.C., Lin C.J. (2011). LIBSVM: A library for support vector machines. ACM Trans. Intell. Syst. Technol..

[42] Liu X., Dou Y., Yin J., Wang L., Zhu E. Multiple Kernel k-Means Clustering with Matrix-Induced Regularization. Proceedings of the AAAI 2016.

